# Identification of Lighting Strike Damage and Prediction of Residual Strength of Carbon Fiber-Reinforced Polymer Laminates Using a Machine Learning Approach

**DOI:** 10.3390/polym17020180

**Published:** 2025-01-13

**Authors:** Rui-Zi Dong, Yin Fan, Jiapeng Bian, Zhili Chen

**Affiliations:** School of Aeronautics and Astronautics, Shanghai Jiao Tong University, Shanghai 200240, China; dongruizi@sjtu.edu.cn (R.-Z.D.); jiapengbian@sjtu.edu.cn (J.B.); czl323@sjtu.edu.cn (Z.C.)

**Keywords:** lightning strike damage, carbon fiber-reinforced polymer, machine learning, residual strength, composites, identification, prediction

## Abstract

Due to the complex and uncertain physics of lightning strike on carbon fiber-reinforced polymer (CFRP) laminates, conventional numerical simulation methods for assessing the residual strength of lightning-damaged CFRP laminates are highly time-consuming and far from pretty. To overcome these challenges, this study proposes a new prediction method for the residual strength of CFRP laminates based on machine learning. A diverse dataset is acquired and augmented from photographs of lightning strike damage areas, C-scan images, mechanical performance data, layup details, and lightning current parameters. Original lightning strike images, preprocessed with the Sobel operator for edge enhancement, are fed into a UNet neural network using four channels to detect damaged areas. These identified areas, along with lightning parameters and layup details, are inputs for a neural network predicting the damage depth in CFRP laminates. Due to its close relation to residual strength, damage depth is then used to estimate the residual strength of lightning-damaged CFRP laminates. The effectiveness of the current method is confirmed, with the mean Intersection over Union (mIoU) achieving over 93% for damage identification, the Mean Absolute Error (MAE) reducing to 5.4% for damage depth prediction, and the Mean Relative Error (MRE) reducing to 7.6% for residual strength prediction, respectively.

## 1. Introduction

Composite materials, such as CFRP, are favored for their high strength-to-weight and stiffness-to-weight ratios, leading to widespread applications in fields such as aerospace structures, pressure vessels, and automotive components [1]. The extent to which these composite materials are applied has become a key indicator of the advancement of aircraft technology [2]. It is reported that commercial aircraft may face a lightning strike event annually [3]. The aircraft structures made by CFRP laminates (composite material consisting of carbon fibers and polymer resin, typically epoxy), however, are particularly susceptible to high-energy lightning strike events due to their relatively low electrical and thermal conductivities [4]. When a CFRP laminated structure is struck by lightning, more resistive heating is generated due to its higher resistivity compared to metals. This leads to severe thermal ablation damage, significantly reducing the local stiffness and strength of the components. Ultimately, it can cause serious damage to the CFRP laminated structure and endangers the safety of aircraft flight [5,6,7]. Therefore, research into methods for predicting lightning strike damage (destruction or injury caused by a lightning strike) to aircraft composite laminate is of significant importance.

Although CFRPs exhibit excellent performance, their poor impact resistance necessitates special attention to their mechanical properties after impact in practical applications [8]. Damage in CFRP laminates under impact typically manifests in two primary forms: intra-laminar damage (including matrix cracking, fiber failure, and fiber–matrix debonding) and interlaminar damage (delamination), with pit depth primarily reflecting the state of intra-laminar damage [9,10]. The unique laminated structure of composite materials can lead to significant performance degradation due to internal structural damage incurred from impacts [10]. Studies indicate that such internal damage can reduce the load-bearing capacity of composite structures by over 50% [11]. Therefore, pit depth is crucial for assessing the extent of damage in composite materials. Additionally, the damaged area is an important indicator of the overall damage condition of composite materials, mainly reflecting the extent and distribution of the damage [12]. This assessment method is also applicable to analyzing damage caused by environmental factors such as lightning strikes. After suffering lightning strikes, CFRP laminates are usually required to be assessed for the extent of damage by examining the damaged area and depth [13], making these two parameters essential for evaluating the severity of lightning strike damage. Moreover, there is an obvious inverse relationship in statistics between the area of damage and damage depth and the residual strength (the remaining load-bearing capacity of a material or structure after experiencing damage or failure) of the CFRP laminates. The larger the area of damage and the deeper the damage, the lower the residual strength of the material [9,14]. This relationship indicates that measuring the damage area and depth not only provides a visual description of the damage characteristics but also serves as a key factor in assessing the overall performance degradation of CFRP laminates. Therefore, by quantitatively analyzing the damage area and depth, one can more accurately assess the structural integrity and load-bearing capacity of composite materials after a lightning strike, providing an important basis for formulating repair strategies and optimizing design.

In recent years, research on the residual strength of CFRP laminates after lightning damage has made significant advances through numerical simulations. Considering the loading conditions, modeling approaches can be classified into thermal–electric [15,16,17,18,19,20,21,22,23,24,25,26], thermal–electric–pyrolytic [22,25,26,27,28,29,30,31,32], and thermal–electric–structural [33,34,35,36] analyses. Specifically, Millen [37] proposed a virtual testing framework to predict the residual compressive strength after lightning strikes and discussed the predicted results of thermal and mechanical damage. Experimental and simulation results showed good consistency, providing valuable validation for CAL test design. Dhanya and Yerramalli [38] employed a finite element-based electrothermal–mechanical coupled model to simulate the lightning effects under different peak currents and predicted the residual compressive strength of CFRPC. Li et al. [27] developed a numerical analysis program that included lightning strike ablation damage simulation and residual compressive strength calculation modules. Simulation results indicated that laminates with TSSC protection and 12-layer interlaminar CNF protection significantly improved the residual compressive strength. The main compression damage types in the laminates after lightning strikes were fiber–matrix shear damage and fiber fracture damage. However, these methods typically involved high temporal and financial costs [39]. Finite element models attempt to simulate the complex multi-phase interaction mechanisms between composite materials and lightning strikes. Due to the unresolved complexity of these mechanisms, even the most advanced numerical models struggle to encompass all relevant factors fully. Additionally, solving complex multi-physics equations presents challenges in numerical convergence and high computational costs. Numerical simulations require precise material parameters, which are temperature-dependent. Although some studies have predicted these characteristics using theoretical approaches like thermodynamics, predictions across different research groups were inconsistent [4]. Therefore, the adjustment and optimization of finite element models are highly complex and time-consuming, adding difficulty and uncertainty associated with studying the residual strength of CFRP laminates following lightning strike damage.

In the field of lightning strike testing, research primarily focuses on the following aspects. Hirano et al. [40] conducted 40 experimental tests to investigate the influence of three factors—lightning current waveforms, current parameters, and specimen thickness—on damage outcomes. They identified three distinct damage modes: fiber breakage, resin degradation, and delamination. Fiber breakage was attributed to shock waves generated by the supersonic expansion of ionized channels, resin degradation resulted from the dielectric breakdown of air and resistive heating, and delamination was caused by interlayer explosions during matrix combustion and pyrolysis. Their results demonstrated that specimen size and thickness had minimal influence on lightning strike damage. However, the fiber damage area and maximum damage depth showed a positive correlation with the lightning current peak value, the resin damage area correlated positively with the lightning charge *Q*, and the delamination area was associated with the integral of the lightning current duration. Feraboli and Miller [41] investigated the lightning strike damage behavior of HTA/7714A composite laminates with and without bolts. Damage regions and morphologies were assessed using ultrasonic C-scans and optical microscopy. The study revealed that under high-intensity lightning currents, damage in composites was predominantly caused by thermal–mechanical shocks from ultrahigh-speed plasma expansion, resistive heating, and vaporization recoil effects. For bolted composite laminates, damage propagated rapidly and penetrated nearly the entire laminate thickness, whereas for unbolted laminates, damage was concentrated in the top 2–4 layers. In a subsequent study, Feraboli and Kawakami [42] compared the damage mechanisms of composite laminates under mechanical impact and simulated a lightning current impact. The results indicated that the energy dissipation rate within the composite under a lightning current impact was significantly higher than under a mechanical impact. However, the extent of damage in terms of area and residual strength was less severe under lightning strike conditions than under mechanical impact. Ben et al. [43] conducted artificial lightning strike experiments to investigate the damage mechanisms of CFRP under lightning strikes. Using high-speed cameras and infrared thermal imaging technology, they observed both the lightning strike process and the pyrolysis behavior. Through the analysis of the lightning process and damage patterns, the authors elucidated the damage behaviors of carbon fiber-reinforced composites. The lightning-induced damage was categorized into fiber breakage, delamination, fiber sublimation, and resin decomposition. Delamination was primarily caused by the expansion pressure from pyrolytic gases, which was mainly influenced by the fiber orientation of the layers above the interface. Fiber sublimation and resin decomposition resulted from the heat generated by Joule heating. Soulas et al. [44] introduced an alternative lightning strike testing method based on energy deposition, using spherical impact tests to simulate lightning strikes. They compared damage area and depth distribution between the two approaches on composite laminates. The results indicated that equivalent spherical impacts produced surface delamination damage distributions similar to those from lightning strikes. However, lightning-induced delamination was concentrated in the front half of the laminate thickness, while spherical impacts caused damage primarily in the rear half. Lightning strike tests provide direct damage data by simulating lightning strikes on composite materials. These tests are typically used to summarize experimental phenomena and experiences and can also assess the accuracy and applicability of numerical simulation models. However, lightning strike testing often requires expensive equipment and materials, which limits the repeatability and scalability of the experiments [4]. Due to high experimental costs and operational complexity, it is challenging to frequently repeat tests to gather large quantities of data, further complicating the achievement of high-precision and consistent results during experimental validation [45]. However, with the development of artificial intelligence (AI) technology, the AI-based data analysis of experimental data has emerged as a novel approach. By mining and learning from extensive experimental data, more accurate predictive models can be established, providing a new analytical tool for evaluating the mechanical performance of materials under lightning strike conditions.

A substantial body of literature has demonstrated the significant role of artificial intelligence (AI) technologies in predicting the mechanical properties of composite materials [46,47,48,49,50]. The physics-guided convolutional neural network (PGCNN) integrated physical and data-driven models to predict fatigue damage in CFRP laminates by detecting changes in the power spectral density of Lamb wave signals and structural stiffness degradation [51]. By using an addressable conducting network (ACN), combined with a resistance network model based on Kirchhoff’s law and a finite element analysis, data for deep learning were generated. An artificial neural network (ANN) was then used for damage detection and evaluation, predicting the mechanical properties of CFRP laminates [52]. By combining AI with infrared thermography, deep neural networks were used to detect and segment the impact damage on CFRP laminates, predicting their mechanical properties. The models were trained on mid-wave and long-wave infrared images, achieving high F1 scores [53]. Surface profile and internal damage data of CFRP laminates were obtained through low-velocity impact tests and machine learning models were used to predict impactor shapes, delamination areas, and lengths. The feasibility of inferring invisible internal damage from surface damage profiles was verified using ridge regression and random forest models [54]. An ANN was developed to predict the lateral-torsional buckling resistance of slender steel cellular beams. This model accurately estimated the resistance and relevant buckling modes, making it a viable design tool [55].

Although the recent study carried out by Millen et al. [56] has highlighted the potential of applying machine learning to predict thermal damage in composite laminates caused by lightning strikes, there has been no exploration of how to estimate the residual strength of carbon fiber composite laminates after lightning damage. The application of deep learning in the field of lightning strike damage remains limited, yet it holds significant potential for future advancements. Following an impact, the residual strength of CFRP laminates decreases as the impact energy increases [57]. Compared to conventional numerical simulations, AI-based prediction of CFRP properties, which relies on real datasets and abstracts away from physical models, typically offers faster computational speeds, thereby enhancing the efficiency and feasibility of lightning damage prediction. Deep learning excels at comprehending intricate nonlinear relationships within datasets, thus providing a more nuanced understanding of how lightning events impact CFRP laminates. Furthermore, AI technology can automatically extract features without reliance on manually set model parameters, reducing the need for specialized knowledge and decreasing the subjectivity of the model [58]. By applying AI techniques to predict the residual strength of CFRP laminates following lightning strike, the severity of the impact and the post-damage structural integrity can be accurately assessed, effectively mitigating safety risks and ensuring flight safety. This predictive approach also enables airlines to perform targeted repairs based on the actual extent of damage, avoiding unnecessary overhauls and reducing maintenance costs and time. Consequently, this method enhances the efficiency and cost-effectiveness of aircraft maintenance.

Therefore, this study proposes an innovative machine learning-based approach to predict the residual strength of CFRP laminates after a lightning strike. The UNet neural network is particularly effective in processing images to identify and quantify lightning-affected areas in CFRP laminates, thereby enabling an accurate visualization of the damaged regions. The damage area, along with relevant lightning and mechanical parameters, is input into two separate, fully connected neural network (FCNN) models for predicting damage depth and residual strength, respectively. Experimental results indicate that both damage depth and area are essential for evaluating the residual strength of CFRP laminates. Accordingly, this study introduces damage depth as a new input parameter, along with damage area, lightning, and mechanical parameters, to improve the accuracy of residual strength predictions. This integrated approach provides a relatively reliable basis for assessing the structural integrity of CFRP laminates following a lightning strike. The main objectives are summarized as follows:

1. This study is the first attempt in the field of lightning damage assessment to propose the application of AI for identifying damaged areas and using damage depth and area as key parameters to predict residual strength. This approach offers an efficient, rapid, and relatively accurate tool for assessing lightning damage in aircraft.
2. To enhance the accuracy of identifying lightning strike damage regions and their areas, this study integrates the UNet neural network with the Sobel operator.
3. Experimental results indicate a clear linear relationship between the increase in damage area or depth and the decrease in the compressive residual strength of CFRP laminates, making these two parameters key indicators for accurately evaluating structural integrity and residual strength.
4. To estimate damage areas, this study analyzes images of the affected regions combined with structural and/or lightning parameters, using two fully connected neural network (FCNN) models to separately estimate damage depth and residual strength. The model predicting residual strength incorporates both damage depth and area as input parameters.

## 2. Materials and Methods

**Figure 1:**
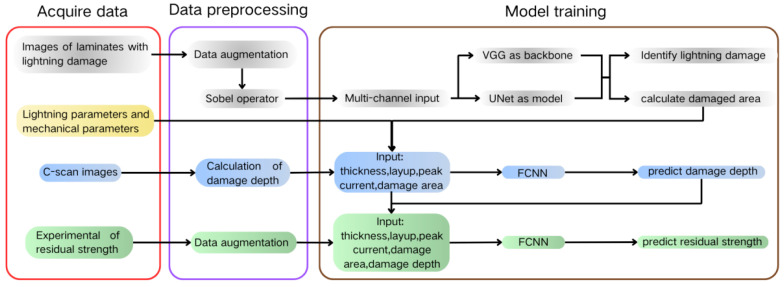
Flowchart of the overall methodology.

This study primarily focused on identifying lightning strike damage and outputting the damage area. Additionally, a two-step method was developed to predict the residual strength of lightning strike damage based on damage area, depth, and other parameters. The detailed process flow is shown in Figure 1.

### 2.1. Dataset Acquisition

**Figure 2:**
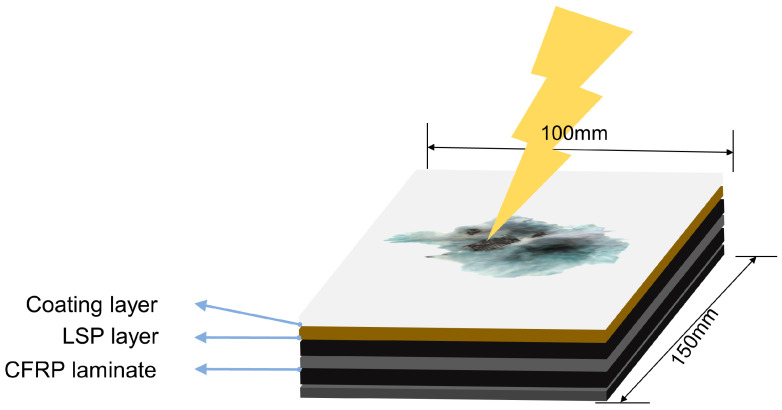
Schematic diagram of a CFRP sample used in the lightning strike experiment.

To acquire datasets for identifying lightning strike damage areas and predicting the damage depth and residual strength of lightning strike damage in CFRP laminates, this study employed a comprehensive approach to collect multiple data points after lightning strike damage. The datasets used in this research were based on experiments involving CFRP laminates subjected to lightning strikes, as shown in Figure 2. A typical sample contained a coating layer (CL), lightning strike protection (LSP) layer, and CFRP laminate. The collected data encompassed photographs of the damage area, C-scan imaging, mechanical performance data, layup data of the CFRP laminates, and lightning parameters, resulting in an extensive and detailed dataset.

#### 2.1.1. Image Processing

The dataset for identifying lightning strike damage areas on CFRP laminates was manually collected, primarily sourced from simulated lightning strike experiments on CFRP laminates. The dataset included high-quality damage images captured from the samples after the lightning strike experiments. Based on the experimental results, a total of 197 images of lightning strike damage to CFRP laminates were captured. It is particularly important to note that these 197 images were from different samples, meaning that 197 distinct CFRP samples were subjected to lightning strike testing, and each sample had corresponding images taken. To ensure the quality and applicability of the dataset, this study adopted strict methods to ensure the reliability and accuracy of the data. Photos of the damaged areas were taken from fixed positions and angles to ensure that samples were fully represented in the photographs. Only samples where the damaged area was less than the total area of the composite laminates were selected to ensure accurate residual strength values. Moreover, all damaged (CFRP laminates fracture or actual area of damage far exceeding the surface area of the laminate), duplicate (multiple pictures of the same CFRP laminate), irrelevant (with no significant lightning damage), and blurry (refer to poorly captured photographs caused by human factors) images were excluded. This ensured that the resulting dataset was highly authentic and representative. The image dataset acquired in the experiment was subject to certain data errors due to its manual acquisition. Furthermore, to ensure data quality, various potential damage scenarios that could occur in real experiments were excluded, which introduced certain limitations to the dataset.

**Figure 3:**
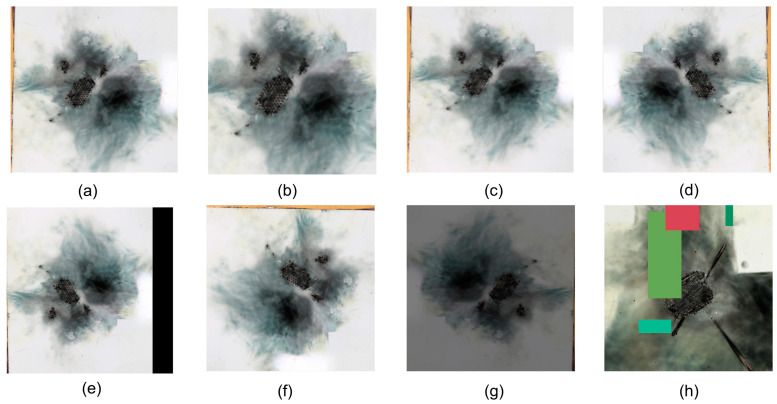
Comparative images of lightning damage samples: (**a**) original image, (**b**) cropping, (**c**) adding Noise, (**d**) mirroring, (**e**) translation, (**f**) rotation, (**g**) changing brightness, and (**h**) mosaic augmentation (the colored rectangular blocks are randomly generated mosaic occlusions designed to enhance the complexity of images).

Ultimately, 165 images of CFRP laminates lightning strike damage were available for neural network learning with 96 dpi horizontal and vertical resolution. However, this number was significantly smaller compared to the thousands to tens of thousands of images in mature datasets, which could lead to overfitting during training and make it unsuitable for direct use in training object detection networks. To address this challenge, various data augmentation techniques were employed to expand the dataset to 1987 images and divide it according to a training/validation/test set ratio of 6:2:2. For specific data, refer to Table 1. Data augmentation techniques fell into two main categories: geometric transformations and color transformations. Geometric transformations included translation, cropping, rotation, mirroring, and adding noise, which helped simulate variations in images across different scales, positions, and perspectives. Color transformations involved only adjusting the image’s brightness to obtain the images under different lighting and color conditions. The goal was to simulate the behavior of the laminate under different lighting scenarios, thereby diversifying the environment in which the images were captured. Additionally, the mosaic data augmentation technique was also introduced. By applying random rotations, flips, cropping, Gaussian blurring, color changes, and adding mosaic obstructions, the background was enriched, and more complex environments were simulated. The parameters for data augmentation are provided in the Supplementary Materials, and comparison images of lightning strike damage samples can be found in Figure 3. This approach, by increasing data volume, improving data quality, and enhancing data diversity, helped enhance the model’s robustness and learning capabilities, thereby effectively preventing overfitting. During the training process, the primary purpose of applying the above data augmentation techniques was to simulate lightning strike damage under various environments and conditions, thereby enhancing the accuracy of identifying real lightning strike damage images.

**Figure 4:**
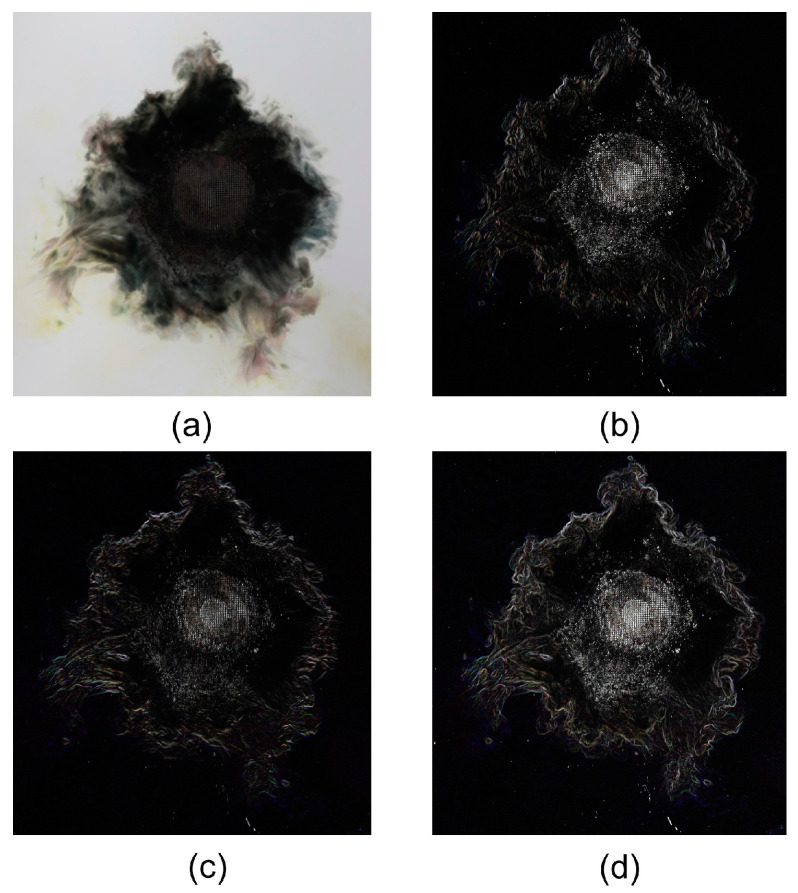
Sobel operator preprocessing of an image of a lightning strike-damaged CFRP laminate: (**a**) original image, (**b**) horizontal gradient image, (**c**) vertical gradient image, and (**d**) combined gradient image.

**Figure 5:**
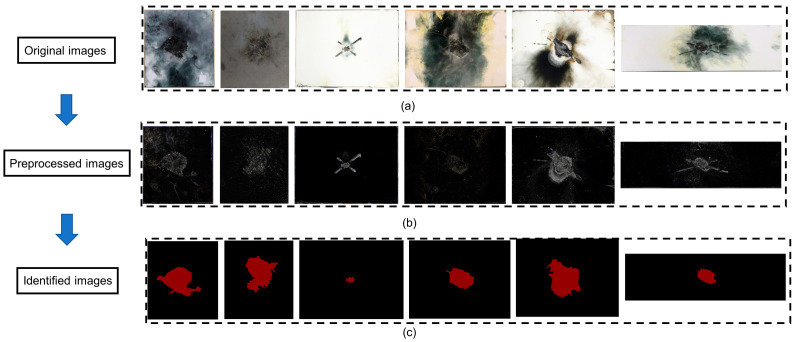
Original, preprocessed, and damage-identified images. (**a**) original images of typical lightning strike damage, (**b**) images after Sobel operator preprocessing, and (**c**) images of damage areas identified by the neural network.

The complexity and diversity of lightning strike damage in CFRP laminates is unique due to the high-energy impacts produced by lightning. The variety of damage types includes, but is not limited to, surface cracking, perforation, fiber breakage, and resin shattering [59]. These damages may exist alone or in mixed forms, and different types of damage may intertwine and affect each other, complicating the analysis and identification of damage. Accordingly, to enhance the accuracy of damage area identification, the Sobel operator preprocessing was employed. The Sobel operator is a commonly used edge detection algorithm that detects edges in images by calculating the gradient of gray levels at each pixel, aiding in the precise localization of damaged areas. The Sobel operator identifies edges in an image by applying specific convolutional kernels, involving gradient calculations in both horizontal and vertical directions. The horizontal gradient component is calculated using the kernel:

(1) $$G_{x}=\left[\begin{array}{c} -1\;\;\;\;0\;\;\;\;1\\ -2\;\;\;\;0\;\;\;\;2\\ -1\;\;\;\;0\;\;\;\;1 \end{array}\right],$$

which emphasizes horizontal changes in the middle row of the image. The vertical gradient component is obtained through the kernel:

(2) $$G_{y}=\left[\begin{array}{ccc} -1 & -2 & -1\\ 0 & 0 & 0\\ 1 & 2 & 1 \end{array}\right],$$

highlighting vertical changes in the middle column. The overall gradient magnitude of the image can be calculated using

(3) $$G=\sqrt{G_{x}^{2}+G_{y}^{2}},$$

thereby revealing the edge locations within the image. The Formulas (1)–(3) reference the work of Sobel et al. (1968) [60]. This effective linear combination of horizontal and vertical gradients produces a clear composite gradient magnitude map. The images after processing them with the Sobel operator are shown in Figure 4. Figure 5 presents a comparison of the original image, the image after Sobel operator preprocessing, and the image identified by the neural network. The original images were all derived from lightning strike experiments. From the comparison, it can be observed that the CFRP laminate’s lightning strike damage areas in the images processed by the Sobel operator highlight the edges and details of the damage. Furthermore, the damage area identified by the neural network accurately marks the specific location and extent of the damage. This combination of techniques not only enhances the visualization of the damage but also improves the accuracy of the neural network in identifying damage within complex environments.

#### 2.1.2. Damage Depth Measure

**Figure 6:**
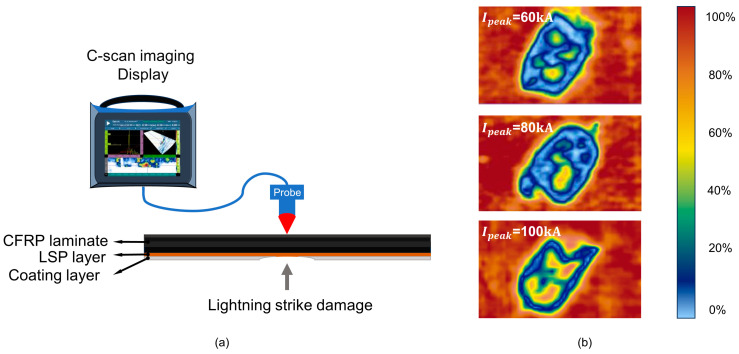
Schematic diagram of C-scan test and mapping images of damages for various injected peak currents. (**a**) The specific principle of conducting a C-scan on CFRP laminates after lightning strike damage and (**b**) Partial results of C-scan images for lightning struck CFRP laminates under different I_peak_ conditions, the transition from red to blue represents the percentage of ultrasound reflections that can be detected.

Each specimen after a lightning strike was inspected by C-scan imaging, with ultrasonic scanning conducted from the backside of the lightning strike point, as illustrated in Figure 6a, and mapping images of the samples subjected to 60 kA, 80 kA, and 100 kA peak current lightning strikes are shown in Figure 6b. By analyzing the energy of the bottom-reflected wave, the location and damage depth can be determined. The dataset consisted of 80 data subsets, with 70 allocated for the training set. Additionally, the prediction model for damage depth established a test set, assigning sample numbers A1 through A10 to that set.

#### 2.1.3. Residual Strength Tests

To comprehensively assess the mechanical performance of CFRP laminates following lightning strikes using waveform A, a series of tensile and compressive residual strength tests were conducted on 165 samples. The objective of these tests was to explore the residual strength performance of various CFRP layup configurations after damage induced by waveform A lightning strikes. However, due to experimental constraints, the available data from tensile residual strength tests were limited and insufficient to construct the dataset required for deep learning applications. Due to the significant deviation of results from special conditions such as end crushing, which were not considered valuable for reference, these outlier data samples were excluded. Experimental data on compressive residual strength were used as the original dataset for the prediction model. As a result, the model did not possess the capability to predict the residual strength of samples exhibiting end crushing. This dataset comprised 80 sets of data, with 70 sets used as the training set. Furthermore, the prediction models for residual strength consisted of a test set with assigned sample numbers A11–A20.

Given the significant limitations in collecting data on the compressive residual strength of CFRP laminates after lightning strikes, to avoid overfitting and enhance prediction accuracy, data augmentation strategies were employed in the experiment, expanding the original training set to 210 samples. These included data perturbation and sample synthesis. Data perturbation involved making minor random adjustments to numerical features in the existing dataset to simulate slight variations that could occur in real-world applications, thereby enhancing the robustness and adaptability of the model. Sample synthesis was performed using the Synthetic Minority Over-sampling Technique for Regression (SMOTER) method to generate new samples not present in the original dataset. This enhanced the adaptability and predictive performance under various layup conditions and damage scenarios. That method adapts the original Synthetic Minority Over-sampling Technique (SMOTE) algorithm to regression tasks by enabling the handling of continuous target variables through the interpolation of attribute values and weighted averaging of the target variable, reflecting a novel approach to handle imbalanced datasets in regression scenarios, and the formula for generating new samples using SMOTER is provided in the study by Torgo et al. (2013) [61]. Assume two samples x_1_ and x_2_, with corresponding target variable values y_1_ and y_2_. The feature values for the new sample x_new_ are generated by interpolation:

(4) $$x_{\mathrm{new}\;}=x_{1}+\gamma \times \left(x_{2}-x_{1}\right)$$

where γ is a randomly generated number in the range [0, 1]. For the target variable y_new_, it is calculated using the following weighted-average formula:

(5) $$y_{\mathrm{new}\;}=\lambda \times y_{1}+\left(1-\lambda \right)\times y_{2}$$

where the weight λ is based on the inverse function of the distances from the synthetic sample to each of the two samples used for synthesis. This can be specifically defined as:

(6) $$\lambda =\frac{d\left(x_{\mathrm{new}},x_{2}\right)}{d\left(x_{\mathrm{new}},x_{1}\right)+d\left(x_{\mathrm{new}},x_{2}\right)}$$

where d(x,y) represents the Euclidean distance between x and y. This calculation method ensures that y_new_ is not merely a simple average of y_1_ and y_2_ but is weighted according to the distances of the new sample in the feature space to x_1_ and x_2_, better reflecting the relationship between the target variable and the features. Employing such data augmentation strategies not only diversified the dataset but also stabilized the training process’s loss curve, making it more accurate. These improvements significantly impacted enhancing the overall predictive performance of the model.

#### 2.1.4. Lightning and Mechanical Parameters

Several studies on CFRP laminates’ lightning strike testing have shown that the electric currents in waveforms B, C, and D are significantly lower than in waveform A, leading to the assumption that waveform A primarily causes most damage [4]. Based on this consensus, waveform A was selected as the experimental waveform for the lightning strike damage experiments on CFRP laminates conducted, and all data pertaining to waveforms used in the related experiments and neural network analyses were exclusively derived from waveform A in this study.

The experiment involved CFRP laminates with various layup configurations having a specific thickness. Each specimen was cut into standardized sizes for compression testing, designed to meet the requirements of the testing apparatus and the objectives of the experiment. All relevant data are compiled in Table 2, which includes but is not limited to the design of the CFRP laminates layup, the thickness of each layup, and the dimensions of the compressive residual strength specimens. The CFRP laminate samples used in the experiments included five different layup schemes listed in Table 2 to investigate the impact of different layup structures on the mechanical performance of CFRP laminates after lightning damage. LSP refers to the lightning strike protection layer present in all CFRP laminates specimens used. Specifically, this involved a copper mesh adhesive film applied to the surface of the CFRP laminates structures, which enhances electrical conductivity and mechanical stability.

### 2.2. A New Method for Identifying Lightning Strike Damage

**Figure 7:**
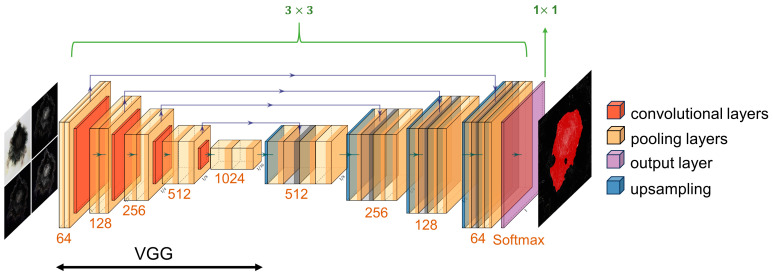
The structure of the UNet neural network for identifying lightning strike damage areas on CFRP laminates.

As shown in Figure 7, the model based on the UNet neural network achieved the identification of lightning strike damage areas on CFRP laminates. In the conventional UNet model, its core structure features a symmetrical encoder–decoder architecture equipped with skip connections to preserve feature information during the encoding process. This design has been widely modified and applied in segmentation tasks in fields such as medical imaging to optimize the capture of details and the utilization of contextual information [62,63,64]. However, the encoder part of the traditional UNet typically employs simple stacked convolutional layers, which may limit the performance of the model in processing complex images. To enhance the feature extraction capability of the UNet encoder, this study adopted the VGG network as the backbone. The VGG network, particularly the VGG-16 model and VGG-19 model, is renowned for its deep convolutional network structure and has demonstrated strong feature extraction and representation capabilities across various image processing tasks [65,66]. Although the dataset for lightning strike damage identification was expanded to 1987 images through data augmentation, it still qualified as a small sample dataset in terms of the scale required for deep learning. To improve training speed and enhance the model’s generalization ability on small sample datasets, a pre-trained VGG network was integrated as the encoder within the UNet architecture. This integration not only utilized the deep feature extraction capability of VGG but also retained the advantages of UNet in image detail reconstruction.

Furthermore, this research innovated upon the UNet neural network by employing a four-channel input strategy as opposed to the conventional single-channel input. This approach included the original image, edge detection responses processed by the Sobel operator along the horizontal and vertical directions, and a linear combination image of responses in both directions. This combination not only preserved the detailed visual information of the original image but also enhanced the representation of edge features, enabling the network to more accurately locate and identify the fine boundaries of damaged areas. In addition, a preprocessing step of adding gray bars was implemented in the UNet architecture, uniformly adding gray bars around the image to adjust its size to meet the network’s input dimensions. This preprocessing step maintained the original proportions and content integrity of the image, preventing computational errors caused by size mismatches, and ensured consistency and accuracy in downstream processing. Moreover, it allowed the model to automatically handle images of arbitrary sizes from different sources without manual intervention. This is particularly important for achieving rapid and efficient automatic detection of lightning strike damage, as it allows images taken on-site or of irregular sizes to be directly fed into the diagnostic system, significantly enhancing the practicality and flexibility in real-world applications.

Regarding the UNet neural network used for identifying the lightning strike damage regions on CFRP laminates, increasing the size of the input images to be 512 × 512 pixels provided more contextual information and aided in feature extraction, within the limits of computer hardware performance and model complexity. The Adam optimizer was used, with an initial learning rate set at 0.0001, which was adjusted according to a cosine decay strategy. The model adopted a phased training strategy to optimize performance. In the initial phase, the model underwent 50 training epochs of frozen training, where the parameters of the network’s backbone were fixed, and the batch size was set to 2. Following this, the model entered a 150-epoch unfreezing phase where all network parameters were involved in training, and the batch size remained unchanged at 2. To monitor training progress and perform model saving, the weights of the model were saved every 5 training epochs, and performance evaluation was conducted to ensure continuous training optimization. This training strategy aimed to gradually adjust network parameters to enhance the final performance while maintaining training efficiency.

### 2.3. A New Method for Predicting Damage Depth and Residual Strength

Damage area and depth, directly reflecting the scale and severity of the damage, are both key characterization parameters for assessing residual mechanical properties of lightning-struck CFRP laminates. A quantitative analysis of the damage area and depth effectively evaluates the structural integrity and load-bearing capacity of CFRP laminates after a lightning strike, playing a significant role in devising repair strategies and optimizing designs.

Although the dataset in this study was relatively small, widely known predictive methods suitable for small samples, such as Gaussian process regression, could not be applied due to the complex and irregular relationships within the data, which would lead to suboptimal prediction results. Thus, a hybrid architecture combining a CNN with an FCNN was developed and validated to estimate the damage depth and residual strength of CFRP laminates. The approach involved using the CNN to process image data and extract spatial features, which were then fed into the FCNN for final prediction. This hybrid architecture leveraged the powerful capabilities of CNNs in image recognition and the efficiency and robust adaptability of FCNNs in handling numerical features.

Due to the correlation between damage area and depth with residual strength, this study devised a two-step neural network approach to predict the residual strength of CFRP laminates. The first model used the total thickness of the CFRP laminates, layup information, and surface damage area as input parameters to construct a model for predicting the damage depth. To more accurately predict mechanical performance, a second model was developed based on the results from the first model to predict the residual strength of CFRP laminates after a lightning strike. The input parameters of this second model included four features, three of which were the same as those used in the dataset of the first model, with the additional feature being the damage depth predicted by the first model. To avoid overfitting, this method employed multiple strategies. The model architecture included several hidden layers, each followed by a Dropout layer to mitigate overfitting. Before training, the features were standardized using the Scikit-learn library’s [67] standard scaler, resulting in data with zero mean and unit variance. This standardization process aided in optimizing the convergence speed and stability of the gradient descent algorithm. During training, the data were input in batches through a data loader built using the PyTorch [68] framework. Additionally, triple cross-validation was implemented to comprehensively assess the model’s performance across different data subsets. The network used the rectified linear unit (ReLU) activation function to add nonlinearity, and the optimizer selected is Stochastic Gradient Descent (SGD), with the Mean Squared Error (MSE) as the loss function, which is the average of the squares of the prediction errors, emphasizing the impact of larger errors. The calculation formula for the MSE is

(7) $$MSE=\frac{1}{n}\sum_{i=1}^{n}\;{\left(y_{i}-{\hat{y}}_{i}\right)}^{2}.$$

where $y_{i}$ is the actual value, ${\hat{y}}_{i}$ is the predicted value, and *n* is the total number of samples. Throughout multiple training epochs, both training and validation losses were monitored and displayed a stable downward trend, indicating that the model was learning the data features effectively and possessed good generalization capabilities.

**Figure 8:**
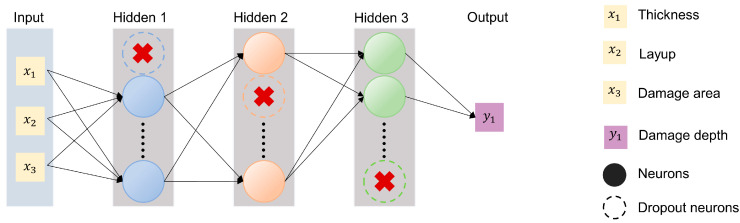
The structure of FCNN for predicting the damage depth in CFRP laminates.

As illustrated in Figure 8, the damage depth in CFRP laminates was predicted through the model incorporating three hidden layers, each accompanied by a Dropout layer with a rate of 0.2 to reduce overfitting. The numbers of neurons in the hidden layers were 15, 10, and 5, respectively. The learning rate was set to 0.0001, and the weight decay coefficient was 0.03. The model underwent 100 training cycles, with the system recording performance on training and validation dataset at the end of each cycle, saving results and handling visualization.

**Figure 9:**
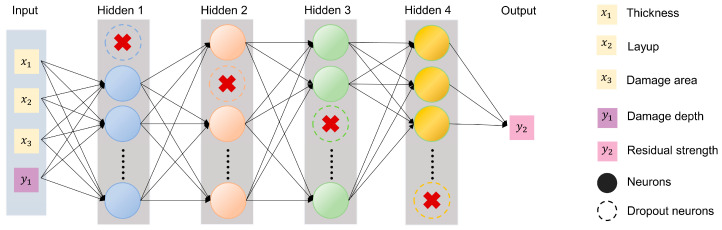
The structure of the FCNN for predicting the residual strength of lightning strike damage in CFRP laminates.

By adding damage depth to the input layer, another regression model, as shown in Figure 9, was utilized to estimate the residual strength of CFRP laminates after a lightning strike. The model contained four hidden layers, each of which was followed by a Dropout layer to reduce overfitting. The numbers of neurons in the four hidden layers were 20, 14, 8, and 4, respectively. Unlike the previous model, this one underwent 120 training cycles, with a learning rate of 0.00001 and a weight decay coefficient of 0.01.

The parameter settings for all models in this study and the reasons for selecting these parameters are summarized in Table 3.

All training and evaluation of deep learning algorithms were conducted in the following experimental environment: a Windows 10 64-bit operating system equipped with an AMD Ryzen 9 3900X 12-Core processor (AMD, Santa Clara, CA, USA), an NVIDIA GeForce RTX 2070 graphics card (NVIDIA, Santa Clara, CA, USA), and 32 GB of memory. This environment utilized Anaconda3-5.3.1 (Anaconda Inc., Austin, TX, USA) as the environment management tool, coupled with CUDA 12.2 for GPU acceleration, Python 3.8 as the programming language, and PyTorch 1.12.0 as the deep learning framework.

## 3. Evaluation Metrics

During the evaluation phase of the experiment identifying lightning strike damage areas on CFRP laminates, cross-entropy (CE) loss was employed as the loss function, with mIoU and accuracy designated as the primary evaluation metrics to comprehensively assess the performance of model in the image segmentation task. The mIoU measures the intersection-over-union of the predicted and actual segmentation areas, indicating the accuracy of the model in handling edge details. Accuracy, the most direct performance metric, indicates the proportion of correctly classified pixels relative to the total number of pixels, providing a global perspective on the overall performance. These metrics rigorously evaluate the method’s effectiveness and efficiency. CE loss, a common loss function in machine learning for classification tasks, quantifies the discrepancy between the predicted probability distribution and the actual label’s probability distribution. Since this study involved only the identification of damaged areas in lightning strike images, constituting a binary classification issue, the expression for CE loss could be simplified as follows:

(8) $$CE=-\left(y\log\left(p\right)+\left(1-y\right)\log\left(1-p\right)\right),$$

where *y* is a binary label taking values of 0 or 1, and *p* represents the probability that the model predicts the sample belonging to the positive class *y*. When the probability prediction of the model for the correct category is high (*p* close to 1), the value of the logarithmic component approaches zero, thus resulting in a minimal loss. Conversely, when the probability prediction of model for the correct category is low (*p* close to 0), the logarithmic component becomes significantly large, leading to increased loss. As one of the most crucial performance evaluation metrics in image segmentation tasks, the mIoU primarily measures the similarity between the predicted segmentation areas and the actual segmentation areas. Its advantage lies in its ability to intuitively reflect the capability of the model in handling details, especially in terms of edge alignment. The mathematical expression is as follows:

(9) $$mIoU=\frac{1}{n}\sum_{i=1}^{n}\;\left(\frac{\left|A_{i}\cap B_{i}\right|}{\left|A_{i}\cup B_{i}\right|}\right),$$

where *A_i_* is the area predicted by the model for the *i*th class object, while *B_i_* is the actual area of the *i*th class object, and *n* is the number of categories. Accuracy is the most intuitive performance evaluation metric, reflecting the overall performance of the model across the entire dataset, i.e., the proportion of pixels correctly classified across all categories relative to the total number of pixels. This metric is especially suitable for a quick overview of the overall effectiveness and helps evaluate the feasibility and efficiency of the model in practical applications. The calculation formula for accuracy is

(10) $$A\mathrm{ccuracy}=n_{\mathrm{correct}\;}/n_{\mathrm{total}\;},$$

where *n_correct_* is the number of pixels correctly classified, and *n_total_* is the total number of pixels.

Due to the small numerical differences in the dataset for the damage depth in CFRP laminates, and the values approaching zero when measured in millimeters, it was not suitable to use highly sensitive evaluation metrics for the data. Thus, the MAE, Root-Mean-Square Error (RMSE), and the Coefficient of Determination (R^2^) were used as the primary evaluation metrics during the damage depth prediction evaluation phase. Conversely, as the dataset for the residual strength of CFRP laminates contained larger numerical differences, the MRE, RMSE, and R^2^ were employed instead as the main evaluation metrics for the prediction. These metrics comprehensively assess the performance of the model in regression tasks. The MAE measures the average absolute difference between the predicted values and actual values. The MRE reflects the average proportional error of the predicted values relative to the actual values. The RMSE calculates the average of the squared differences between the predicted values and the actual values. R^2^ measures the goodness of fit of the regression model. These four metrics provide an intuitive and detailed perspective for assessing the performance of the model in handling numerical predictions. The MAE, by directly measuring the average absolute difference between the predicted and actual values, offers a straightforward and intuitive method for assessing the accuracy of the prediction model without overly weighting large errors, and its calculation formula is

(11) $$MAE=\frac{1}{n}\sum_{i=1}^{n}\;\left|y_{i}-{\hat{y}}_{i}\right|.$$

The RMSE amplifies larger errors, making the model more sensitive to outliers or uneven error distributions. It maintains the same units as the data, facilitating an intuitive understanding of the actual impact of errors, and is particularly suitable for evaluating model performance as it aligns directly with the optimization objectives of many machine learning algorithms. The formula for calculating RMSE is as follows:

(12) $$RMSE=\sqrt{\frac{1}{n}\sum_{i=1}^{n}\;{\left(y_{i}-{\hat{y}}_{i}\right)}^{2}}.$$

The MRE is a scale-independent metric that intuitively displays the proportional deviation between predicted values and actual values. It is particularly suitable for sensitively capturing abnormal prediction errors in key areas and assessing the consistency and stability of the model in multi-target predictions. The calculation formula for MRE is

(13) $$MRE=\frac{1}{n}\sum_{i=1}^{n}\;\left|\frac{y_{i}-{\hat{y}}_{i}}{y_{i}}\right|.$$

However, as an evaluation metric, the MRE can exaggerate errors when the actual values are close to zero and data have small numerical differences. Therefore, it should be used in conjunction with other metrics to comprehensively assess model performance. The R^2^ can be expressed as

(14) $$R^{2}=1-\frac{\sum_{i=1}^{n}\;{\left(y_{i}-{\hat{y}}_{i}\right)}^{2}}{\sum_{i=1}^{n}\;{\left(y_{i}-\bar{y}\right)}^{2}},$$

which represents the proportion of the total variation in the dependent variable that is explained by the model, indicating the extent to which the model accounts for the variance in the dependent variable. The value of R^2^ ranges from 0 to 1, where 1 indicates a perfect fit and 0 indicates that the model does not explain any of the variation. Therefore, R^2^ is more suitable for situations where there is a significant difference between actual and predicted values, as it is highly sensitive to the distribution of the data.

## 4. Results and Discussion

### 4.1. Experimental Results

**Figure 10:**
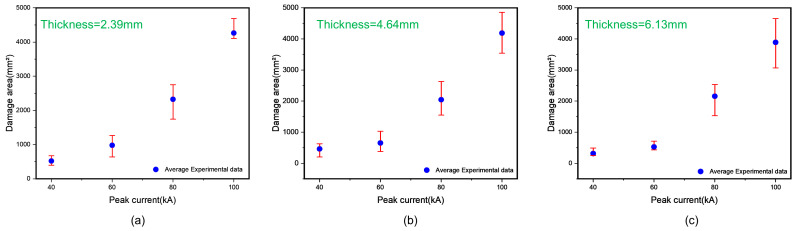
The relationship between the damage area and peak current under the same conditions. (**a**) Thickness = 2.39 mm, (**b**) thickness = 4.64 mm, and (**c**) thickness = 6.13 mm.

In the lightning strike experiments, CFRP laminates samples were subjected to peak currents of 40 kA, 60 kA, 80 kA, and 100 kA to simulate the effects of varying lightning strike intensities on laminate damage. The results, as shown in Figure 10, indicated a consistent trend across different thicknesses: the average damage area was approximately proportional to the peak current. Specifically, under identical conditions, a higher peak current resulted in a larger lightning damage area, and vice versa.

**Figure 11:**
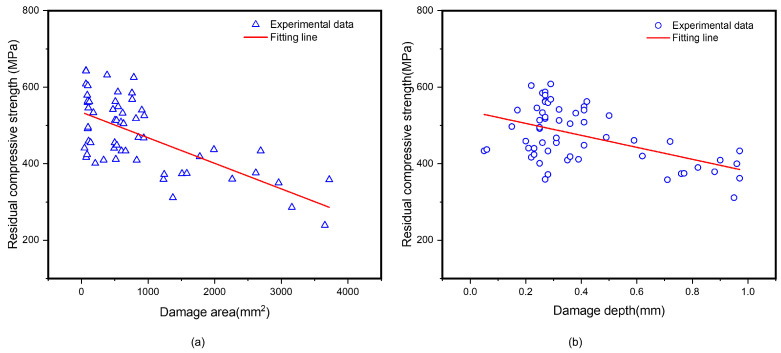
Relationship between the compressive residual strength of CFRP laminates and damage characterization parameters (damage depth and area) under waveform A: (**a**) linear relationships between compressive residual strength and damage area, (**b**) linear relationships between compressive residual strength and damage depth.

Based on the damage detection and residual strength test results of the samples after lightning strikes, we found the linear relationships between the damage characterization parameters and the residual compressive strength values, as illustrated in Figure 11. It can be observed that compressive residual strengths exhibited a clear linear downward trend with the increase in damage area or depth. This trend provided important physical insights for subsequent damage assessment and prediction.

### 4.2. Damage Identification Results

**Figure 12:**
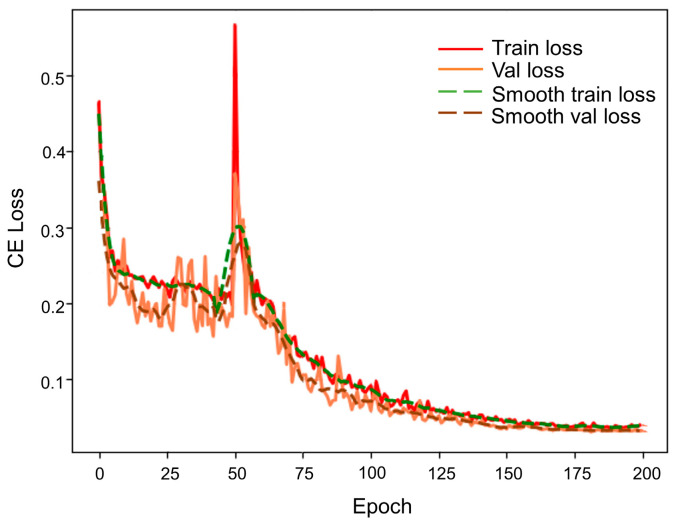
CE loss curve of identifying lightning strike damage areas on CFRP laminates.

In the experiments for identifying lightning strike damage on CFRP laminates, data preprocessing and parameter optimization were conducted. The identification results, as shown in Figure 12, indicated that the CE loss value decreased and stabilized with an increasing number of iterations, while the accuracy increased, becoming stable around 175 epochs. The sudden spike in the CE loss curve at the 50th epoch could be attributed to the unfreezing of previously frozen layers within the network.

### 4.3. Damage Depth Prediction Results

In the prediction of damage depth in CFRP laminates, MSE curves for the model on both the training and validation sets were generated through parameter optimization and data processing. The MSE serves as an assessment metric to measure the deviations between model predictions and actual observations. The training results indicated that the MSE value gradually decreased with an increasing number of iterations and stabilized after the 90th iteration, suggesting that the predictive performance of the model had reached its optimum state after sufficient training.

**Figure 13:**
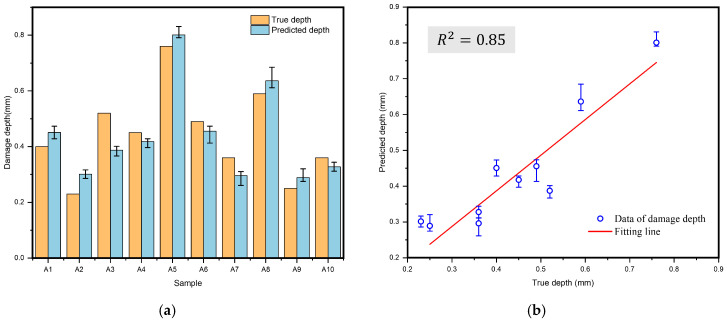
Predicted values and true values for test sets using the model predicting the damage depth in CFRP laminates. (**a**) Bar chart of the true and predicted damage depth for 10 data groups, (**b**) Fitting line between true and predicted damage depth.

To further verify the accuracy of the model, this study set up a test dataset that did not overlap with the training set, comprising 10 data groups. The sample numbers were designated as A1 to A10, with specific layup structures as shown in Table 2. This arrangement was intended to simulate the prediction effectiveness of damage depth under different layup conditions. Five prediction experiments were conducted, and the average values of the predicted damage depths were calculated and illustrated in Figure 13, which also displays the maximum and minimum values from the experiments using error bars. The MAE of the prediction results was only 5.4%, with an R^2^ value of 0.85. After standardizing the data, the calculated RMSE was 0.16, confirming its effectiveness and applicability under specific conditions.

### 4.4. Residual Strength Prediction Results

**Figure 14:**
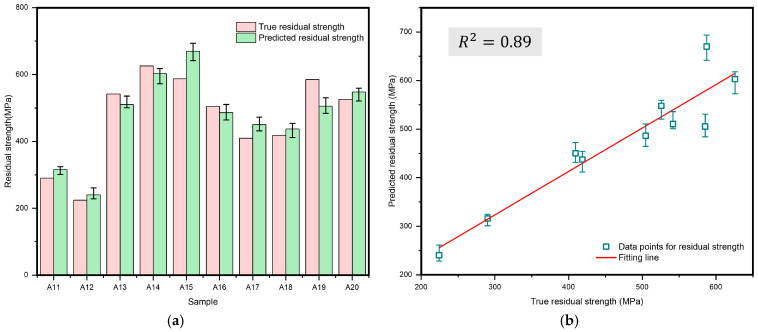
Predicted values and true values for test sets using the model predicting the residual strength of lightning strike damage in CFRP laminates. (**a**) Bar chart of the true residual strength and predicted residual strength for 10 data groups. (**b**) Fitting line between true residual strength and predicted residual strength.

Similarly, to further validate the accuracy of the model, this study established a test dataset that did not overlap with the training set, containing 10 data groups. The samples were labeled from A11 to A20, with the specific layup structures detailed in Table 2, to simulate the prediction effectiveness of CFRP laminates residual strength after lightning strike damage under different layup conditions. Five rounds of prediction experiments were performed, with the mean predicted values of residual strength plotted in Figure 14, which also includes error bars depicting the range between the highest and lowest values recorded during the experiments. The model achieved an MRE of only 7.6%, and R^2^ was 0.89. After standardizing the data, the RMSE was 0.12, confirming its accuracy and applicability under specific conditions.

### 4.5. Discussion

**Figure 15:**
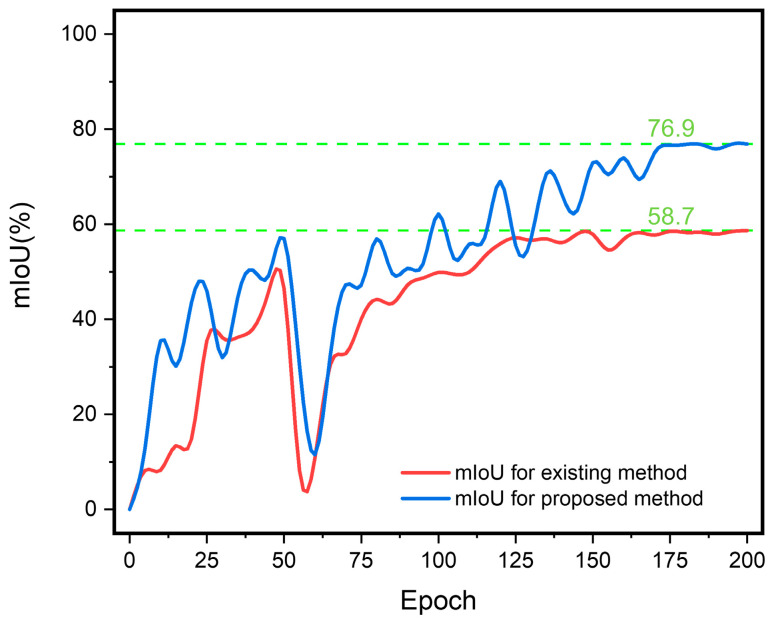
Comparison of mIoU for identifying lightning strike damage areas on CFRP laminates before and after data augmentation.

**Figure 16:**
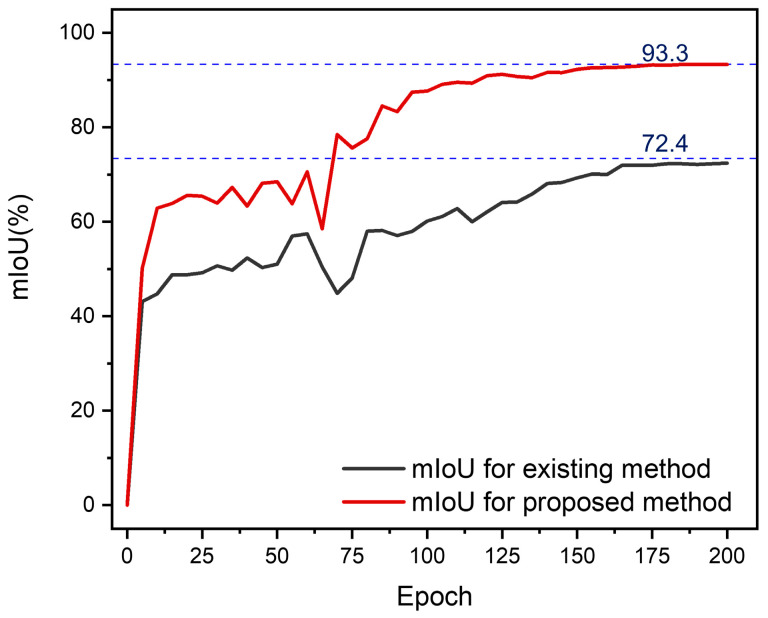
Comparison of mIoU for identifying lightning strike damage areas on CFRP laminates before and after Sobel pretreatment.

Figure 15 illustrates a significant improvement in the model’s accuracy for identifying lightning damage following data augmentation, increasing from 58.7% to 76.9%. Additionally, due to preprocessing with the Sobel operator, the model’s accuracy in detecting lightning-damaged areas significantly improved from 73.4% to 93.3%, as shown in Figure 16.

**Figure 17:**
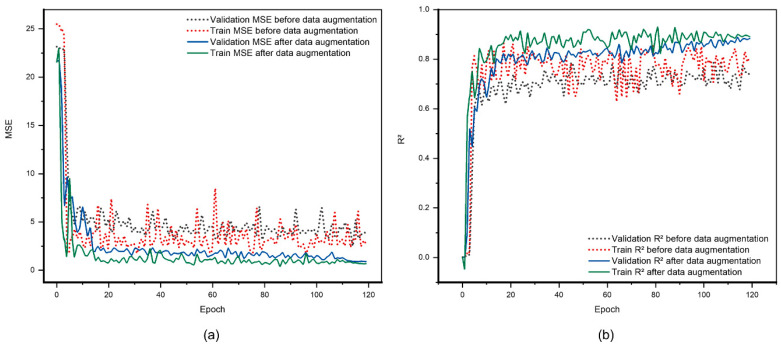
Results of the predictive model for the residual strength after lightning strike damage in CFRP laminates, before and after data augmentation. (**a**) MSE curve results of the predictive model and (**b**) R^2^ curve results of the predictive model.

During the training process of the model predicting the residual strength, MSE and $R^{2}$ curves were plotted for both the training and validation datasets. The MSE was used to measure the error between the predicted values and the actual values of the model, and $R^{2}$ was used to measure the goodness of fit of the model. As depicted in Figure 17, there was a noticeable trend of an MSE reduction as the number of iterations increased. Concurrently, the accuracy of the model progressively improved, culminating in the $R^{2}$ reaching 90%. Additionally, by comparing the data before and after preprocessing, it was seen that the MSE curves for the training and validation sets were smoother and less volatile after preprocessing, and $R^{2}$ significantly improved. Specifically, before preprocessing, the $R^{2}$ for the validation set fluctuated around 81%, while after preprocessing, the $R^{2}$ for the validation set stabilized above 90%, significantly enhancing the ability of the model to predict the performance of CFRP laminates after lightning strike damage. Furthermore, it effectively reduced the extent of overfitting in the model.

**Figure 18:**
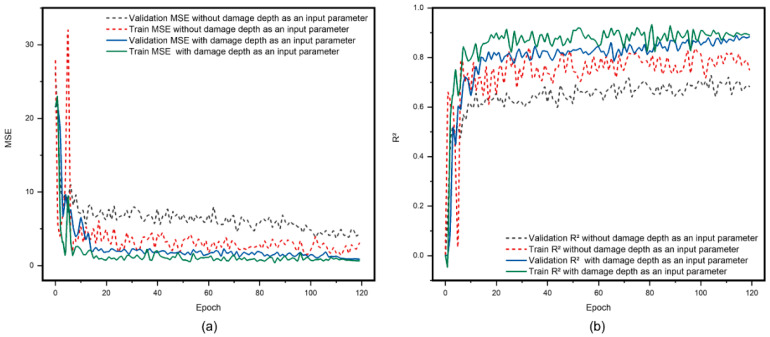
Comparison of the model results predicting the residual strength of CFRP materials after lightning strike damage, with and without considering damage depth as an input parameter. (**a**) MSE curve results of the predictive model and (**b**) R^2^ curve results of the predictive model.

In the study of mechanical properties of CFRP laminates, damage depth is a critical parameter for evaluating structural integrity and residual strength. After a lightning strike, the damage depth directly affects the CFRP laminates’ structural integrity and residual strength, significantly impacting its reliability and safety [69]. Therefore, as proposed in Section 2.3 of this paper, this study used the prediction results of damage depth as an input parameter for the model predicting the residual strength of CFRP laminates. By considering the close relationship between damage depth and residual strength, this approach enhanced the prediction accuracy. The prediction results, as shown in Figure 18, indicated that the predictions of residual strength, when incorporating damage depth as an input parameter, were closer to the actual observations and significantly higher than predictions that did not consider damage depth.

The new method proposed in this study, which used machine learning to predict the residual strength of CFRP, achieved similar accuracy to traditional numerical simulation methods. However, numerical simulations demand a high level of technical expertise from operators, and the results may exhibit substantial variability. In contrast, once trained, the machine learning method is easy to operate and more user-friendly. Compared to relying solely on lightning strike tests, which require considerable time and financial investment to yield conclusions, the proposed method leverages the powerful learning capabilities of machine learning to explore the relationship between various parameters and residual strength based on limited experimental data, achieving higher prediction accuracy at a lower cost.

## 5. Conclusions

This paper introduced an innovative AI method for predicting the mechanical properties of CFRP laminates’ lightning strike damage. This study pioneered applications in the field of lightning damage prediction, expanding the scope of most damage detection research to not only identify damage but also predict the mechanical performance characteristics of the damage. Moreover, this study also demonstrated high accuracy in predicting the damage depth and residual strength of CFRP laminates.

Through the integration of the Sobel operator with a CNN, the study successfully identified and visualized areas damaged by lightning strikes. The combination of the Sobel operator and CNN addressed the common issues of blurred edges and data redundancy in traditional image processing, significantly enhancing the efficiency and accuracy of damage area recognition. Structural and lightning parameters, along with damage area information, were used as inputs to train a deep neural network model that was applied to predict damage depth following lightning strikes. Given that damage depth is a critical indicator of residual strength, it was introduced as a new input parameter into the neural network to predict the residual strength of CFRP laminates. Two predictive models estimated damage depth and residual strength from specific damage images of the CFRP laminates, facilitating the rapid assessment of CFRP laminates’ structural integrity. Extensive experimental validation showed that the model achieved an mIoU of over 93% for identifying damage areas in CFRP laminates.

In the future, the integration of materials science, electrical engineering, and machine learning is expected to evolve into a new technology for predicting lightning strike damage. This technology aims to achieve full automation and intelligence in aircraft lightning strike damage detection. In the aerospace field, residual strength prediction plays a crucial role in developing effective maintenance plans. By understanding the expected performance and service life of components, maintenance schedules can be optimized, thereby reducing the risk of unexpected failures and prioritizing inspections of potentially degraded parts, ultimately improving overall safety. Future developments are anticipated to lead to systems capable of automatically capturing images of aircraft lightning damage, identifying and quantifying damaged areas, and predicting the aircraft’s mechanical performance based on lightning and mechanical parameters reported by sensors. The implementation of such systems will effectively enhance the efficiency and accuracy of aircraft fault detection and maintenance, while significantly reducing costs.

Although this technology shows great potential in the aerospace field, its practical application still faces certain limitations. First, the datasets used in the current research were relatively small, which may limit the generalizability of machine learning models. This is a common challenge in lightning strike experiments. Despite ongoing advancements in lightning strike research, the datasets available remain relatively small compared to other fields and machine learning applications. Moreover, factors such as the materials, number of layers, and the orientation of each layer in composite laminates can affect the damage patterns, depth, and residual strength, potentially limiting the accuracy of damage predictions based on limited samples. Future work could expand the dataset size through additional experimental testing or finite element modeling to study lightning damage to different materials or other types of damage. However, ensuring the reliability of simulation data before using it as input to the model is critical to avoid affecting prediction accuracy. Furthermore, as this technology involves supervised learning, it requires large and precise image annotations for training the model, a process that is both time-consuming and expensive. Therefore, the model should be applied cautiously in all scenarios without further validation and testing. Future research directions should include exploring semi-supervised or unsupervised learning methods to reduce reliance on large, annotated datasets. This would not only lower costs but also improve the applicability and accuracy of the models. The potential applications of this method could extend to health monitoring and the maintenance of vehicles, ships, and other critical structures, thereby advancing technological progress in materials science and structural engineering. Although the current technology shows great promise, further testing and validation are still needed to ensure its effectiveness and reliability in practical applications.

## Figures and Tables

**Table 1:** Detailed information of the image dataset, including image type, number of images, and image resolution.

Image Type	Resolution	Number in Training Set	Number in Validation Set	Number in Test Set	Total Number
Original image	96 dpi	99	33	33	165
Augmented image	96 dpi	1094	364	364	1822
Total image	96 dpi	1193	397	397	1987

**Table 2:** Data of the compressive residual strength training set sample, including layups, quantity, and thickness, along with test set identifiers.

Layup	Total Nominal Thickness/mm	Specimen Size/mm^2^	Quantity in the Training Dataset	Sample No. for Test Dataset of Prediction on Damage Depth	Sample No. for Test Dataset of Prediction on Residual Strength
LSP/[−45/+45/0/+45/−45/90]_2_	2.39	150 × 100	48	A1, A2	A11, A12
LSP/[−45/+45/0/90]_6_	4.64	150 × 100	42	A3, A4	A13, A14
LSP/[−45/+45/0/90]_8_	6.13	150 × 100	39	A5, A6	A15, A16
LSP/[−45/0/+45/90]_6_	4.64	150 × 100	36	A7, A8	A17, A18
LSP/[−45/0/90/+45]_6_	4.64	150 × 100	45	A9, A10	A19, A20

**Table 3:** Hyperparameters and training settings of machine learning models.

Model	Parameter Name	Parameter Quantity	Reason for Parameter
UNet for identifying damage areas	Image size	512 × 512	More contextual information and improve feature extraction
	Learning rate decay strategy	Cosine decay strategy	Stable training and improved model convergence
	Initial learning rate	0.0001	Optimal parameters were selected after multiple debugging sessions
	Batch size	8	
	Epoch	200	
FCNN for predicting the damage depth	Number of hidden layers	3	Optimal parameters were selected after multiple debugging sessions
	Number of neurons	15, 10, 5	
	Learning rate	0.0001	
	Weight decay coefficient	0.03	
	Batch size	4	
	Epoch	100	
FCNN for predicting the residual strength	Number of hidden layers	4	Optimal parameters were selected after multiple debugging sessions
	Number of neurons	20, 14, 8, 4	
	Learning rate	0.00001	
	Weight decay coefficient	0.01	
	Batch size	4	
	Epoch	120	

## Data Availability

Data will be made available on request.

## Supplementary Materials

The following supporting information can be downloaded at: www.mdpi.com/10.3390/polym17020180/s1, Table S1: Specific operations and parameter of data augmenting technology and comparative images of lightning damage samples.
