# Supplementary material for: Identification of Lighting Strike Damage and Prediction of Residual Strength of Carbon Fiber-Reinforced Polymer Laminates Using a Machine Learning Approach
Source: Polymers (Basel). 2025 Jan 13;17(2):180. doi: 10.3390/polym17020180 (PMC11768333; doi:10.3390/polym17020180)
Supplement: Supplementary file 1 [file polymers-17-00180-s001.zip › polymers-3398293-supplementary.pdf]

Tabel S1. Specific operations and parameter of data augmenting technology and comparative images of lightning damage samples.

**Category**	**Specific operation**	**Parameter**	**Comparison between original and augmented images**
Geometric transformation	Cropping	Probability: 0.5<br>Percentage area: 80% of original area	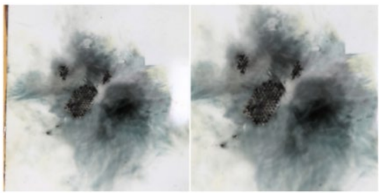
	Adding noise	Probability: 0.4	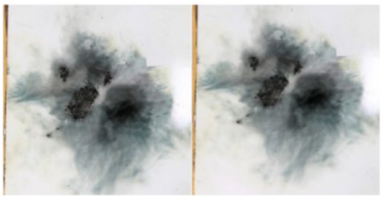
	Mirroring	Probability for horizontal mirroring: 0.5<br>Probability for vertical mirroring: 0.5	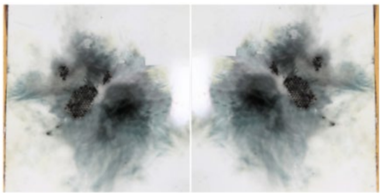
	Translation	Probability: 0.5<br>Magnitude: 0.2	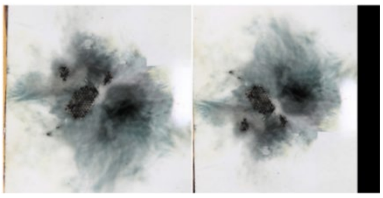
	Rotation	Probability: 0.85<br>Max left rotation: 25 degrees<br>Max right rotation: 10 degrees	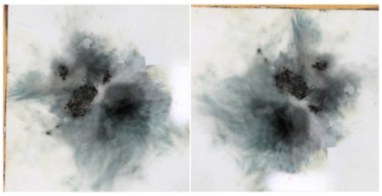
Color transformation	Changing brightness	Probability: 0.5<br>Min factor: 0.7<br>Max factor: 1.3	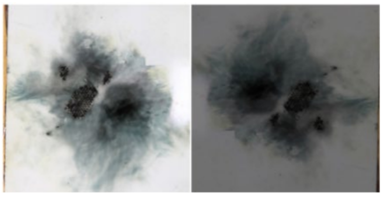
	Mosaic augmentation	Probability: 0.5<br>Mosaic img_size:640<br>Mosaic_border:[-320, -320]	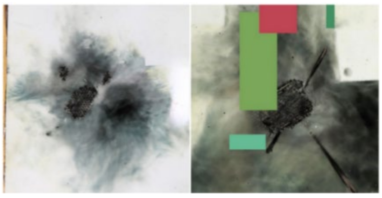
